# Setting the research priorities for pregnancy scanning: a nationally coproduced vision with expectant women, the public and healthcare professionals

**DOI:** 10.1093/bjr/tqaf192

**Published:** 2025-08-04

**Authors:** Jacqueline Matthew, Sharon Watty, Julie Nihouarn Sigurdardottir, Christina Malamateniou, Emily Skelton, Zenab Barry, Mary Rutherford, Sergio A Silverio, Lisa Story

**Affiliations:** Department of Early Life Imaging, King’s College London, London, SE1 7EH, United Kingdom; Department of Clinical Imaging and Medical Physics, Guy’s and St. Thomas’ NHS Foundation Trust, SE1 7EH, United Kingdom; Department of Clinical Imaging and Medical Physics, Guy’s and St. Thomas’ NHS Foundation Trust, SE1 7EH, United Kingdom; Department of Early Life Imaging, King’s College London, London, SE1 7EH, United Kingdom; Department of Forensic and Neurodevelopmental Sciences, King’s College London, London, SE5 8AB, United Kingdom; Division of Midwifery and Radiography, City St. George’s University of London, London, EC1V 0HB, United Kingdom; Division of Midwifery and Radiography, City St. George’s University of London, London, EC1V 0HB, United Kingdom; Department of Early Life Imaging, King’s College London, London, SE1 7EH, United Kingdom; Department of Early Life Imaging, King’s College London, London, SE1 7EH, United Kingdom; Department of Clinical Imaging and Medical Physics, Guy’s and St. Thomas’ NHS Foundation Trust, SE1 7EH, United Kingdom; Department of Psychology, Institute of Population Health, University of Liverpool, Liverpool, L69 3GF, United Kingdom; Department of Early Life Imaging, King’s College London, London, SE1 7EH, United Kingdom

**Keywords:** priority setting partnership, obstetric ultrasound, fetal MRI, sonographers, coproduction

## Abstract

**Objectives:**

Despite sonography studies being integral to routine high quality antenatal care, clinical research in this field is less commonly initiated or led by sonography professionals. It is also unclear what the research priorities are for service users within the UK’s sonography screening and diagnostic pathway.

**Methods:**

Here, we present a national priority setting partnership project, which included 2 surveys, coproduced with the oversight of a stakeholder priority setting partnerships (PSP) group, comprising service users and healthcare professionals, *n = *12.

**Results:**

From the surveys, there were 348 individual responses and 616 validated research questions/uncertainties submitted. The top-ranked 26 indicative questions were discussed at a final joint stakeholder meeting (*n = *17) and the top 10 research priorities for pregnancy scanning research were voted for and agreed by consensus. These fell into 6 main themes: (1) maternal and parental experience; (2) emerging technology; (3) screening, prediction, and diagnosis, (4) role of pregnancy MRI, (5) continued professional development, training and education, and (6) service delivery and workforce.

**Conclusions:**

We envisage this PSP will support teams of researchers and clinicians with important and achievable targets to move the field of pregnancy scanning forward.

**Advances in knowledge:**

This is the first coproduced antenatal scanning priority setting partnership highlighting real world issues and lines of enquiry as proposed by service users and health care professionals.

## Introduction

Antenatal imaging plays a critical role in fetal and maternal screening, diagnosis, treatment, and management during pregnancy and in addition, reflects landmark moments in a woman’s pregnancy journey.^1–3^ To date, there have been no priority setting partnerships (PSP) for pregnancy imaging research. PSPs enable clinicians, patients, and carers to work together to identify and prioritize which questions in particular areas of health and care could be answered by research.^4^ Established in 2004, the James Lind Alliance (JLA) sought to address the mismatch between research activity and the questions that patients and clinicians have.^5^ Medical doctors, nurses, midwives, and allied health professionals to include radiographers and sonographers, are uniquely placed to develop research capacity and capability within the NHS and to lead changes in practice and services.^6^^,^^7^ To date, there have been 12 partnership activities relating to women’s health, with limited themes related antenatal imaging.^8^ PSPs are commonly related to disease specific conditions, however, there are examples of discipline specific priority exercises in physiotherapy and occupational therapy.^9^^,^^10^ A 2017 Delphi consensus study was conducted by the UK’s College of Radiographers and aimed at prioritizing research topics for the radiographic profession.^11^ This consensus study primarily involved clinical practitioners, and, because radiography has a wide range of specialisms providing screening, diagnostic, and therapeutic services for many important health conditions, none of the top 10 priorities were specifically related to ultrasound services or pregnancy.

Maternity faces increased evidence that Black, Asian, and other minority ethnic women are experiencing higher levels of mortality and morbidity during pregnancy^12^ and are subjected to greater levels of racial discrimination.^13^ Minoritized communities may also find healthcare harder to access,^12^^,^^13^ may struggle to navigate the healthcare service, particularly if facing language barriers,^14^^,^^15^ which, may also inadvertently lead to them being excluded from pregnancy research.^16^ Therefore the aim of this project was to coproduce a list of the most important research priorities curated by women, the public, and health professionals on the topic of pregnancy scanning research with a particular focus on ethnic minority engagement. With this ‘pregnancy scanning PSP’ we hoped to provide a useful resource not only to students, service managers, educationalists and clinical academics, but importantly to practicing healthcare professionals (HCPs) involved in pregnancy scanning research.

## Methods

### Design and analysis

**Figure 1.**
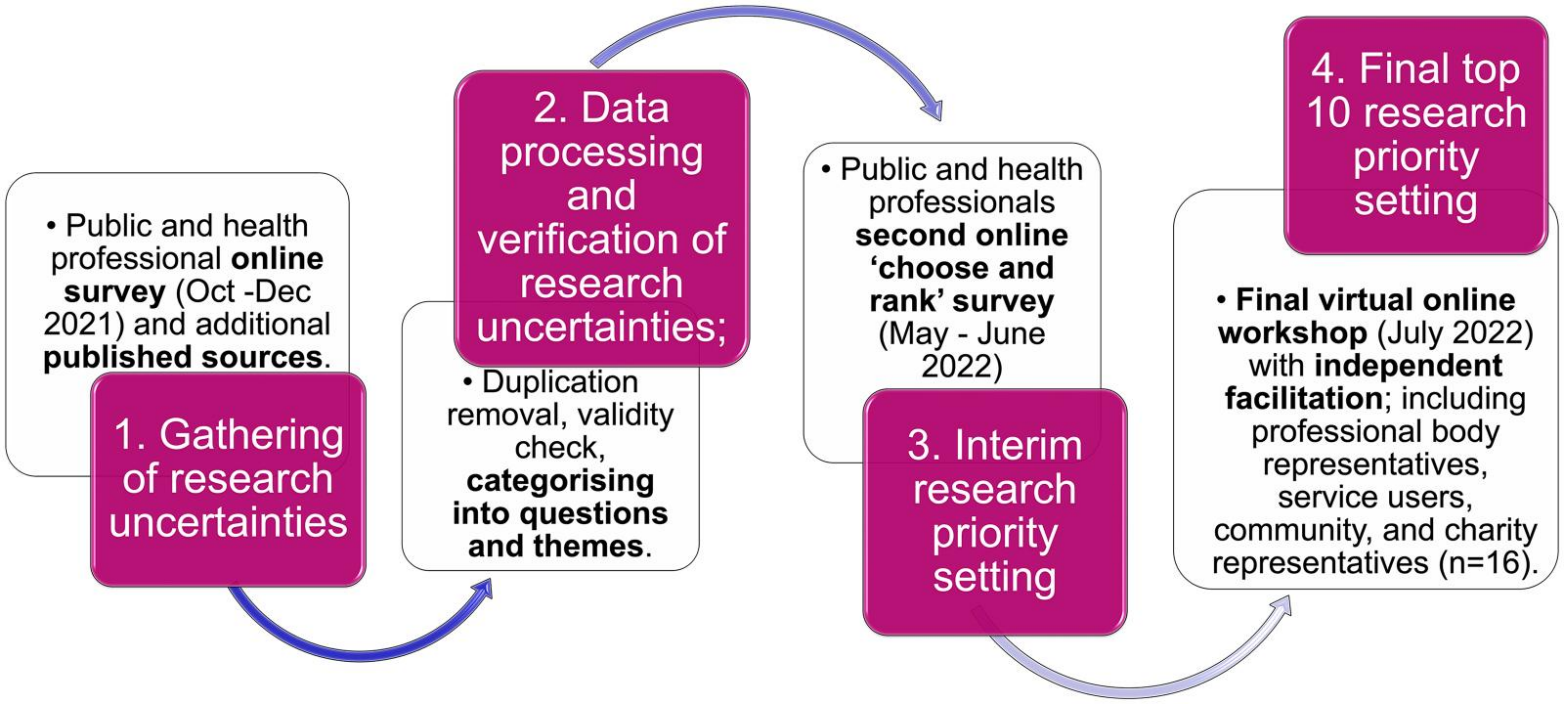
Priority setting partnership four step process.

The PSP followed a 4-step modified National Institute for Healthcare Research JLA method which included a national survey, regular meetings with a PSP stakeholder group during the entire process (Figure 1). This group included 12 members, with 6 meetings taking place during the course of the investigation over 12 months. The group spanned service users, with recent experience of antenatal care, and HCPs, including sonographers, a psychologist with expertise in women’s health, a fetal medicine specialist, and a midwife. Ethics advice was sought from King’s College London research ethics committee for the PSP survey, who advised that, as a public engagement exercise, no ethical approval was required. Nonetheless, the PSP survey was nested in a larger research project which gained ethical approval (research ethics number: HR/DP-20/21-21756). All survey respondents had the opportunity to provide basic demographic data as part of the PSP to include gender, location, and HCP background.

As an overview, the national PSP survey was advertised between October and December 2021 via social media (eg, Facebook, Instagram, X [formerly Twitter], Mumsnet), professional networks (Society of Radiographers, British Medical Ultrasound Society), charities (Antenatal Results and Choices, Better Births), and relevant grassroots organizations (Maternity voices partnerships, Five Times More). In addition, research questions, also referred to as “uncertainties” in this context due to the nature of submissions, were collected from a public dialogue focus group project about scanning and surgical innovations in pregnancy,^17^ from qualitative semistructured interviews about antenatal scans from the Healthtalk resource by the Dipex charity,^18^ and a literature review of existing published PSP about related fields or conditions in pregnancy.^11^^,^^19–36^

STEP 1: Collecting research uncertainties from a range of sources. The PSP questions were formulated in collaboration with the stakeholder group. These were designed to ask the public and HCPs for their research queries related to antenatal imaging, by answering these four open-ended questions:

1. Do you have any suggestions for future research questions related to very early pregnancy (ie, preconception to 16 weeks of pregnancy)?
2. Do you have any suggestions for future research questions related to mid or late pregnancy (ie, from 16 weeks to term)?
3. Do you have any suggestions for research questions related to advanced antenatal scans, for example, 3D/4D ultrasound, pregnancy MRI or other computing, software or engineering technologies or innovations (including artificial intelligence)?
4. When thinking about patient/parent care or staff and scanning services, do you have any suggestions for research questions about accessibility, service delivery or the workforce?.

STEP 2: Processing and verifying the submitted research uncertainties from step 1. This involved removing out of scope and non-questions, then transforming the valid questions into final indicative questions.

To maintain their essence, the valid submitted questions were given 1 or 2 thematic codes to describe the questions, and the level of ambiguity was recorded (high or low).^37^ A single researcher completed the initial coding and indicative question list (S.W.). Then a sample of submitted questions was processed by two additional researchers (J.M., L.S.) to assess agreement—any disagreements were discussed in a consensus meeting and a final conclusion agreed between the 3 researchers. Finally, the initial long list of indicative questions were then consolidated by way of a joint discussion with the 3 researchers and the PSP stakeholder group, that is, removing duplications and refining wording of the indicative questions.

STEP 3: An interim “rank and choose” survey. The short list of questions was circulated via second national survey to participants of the first survey. In the “ranking” stage, each respondent ranked the priority level of each question (high, medium, low, not sure). In the “choose” stage of the survey, the respondent were shown their highest priority questions, organized under relevant themes, and then asked to give one vote for the most important. The indicative questions were given a weighted score based on number of high priority rankings and votes as the most important in order to arrive at a top 25 priority list.

STEP 4: Final top 10 chosen. The final stage in the process involved a wider stakeholder group including charities, additional HCP leaders, and service users, to discuss the top 25 results of the interim survey and to vote on the top 10 highest priorities.

### Workshop

Seventeen stakeholders took part in a 3-hour virtual workshop organized and led by professional external facilitators (3KQ) in collaboration with the project lead (J.M.). The aim was to finalise the top 10 research priorities through discussion and voting. Members of the workshop included; 4 participants with a sonography professional background; 3 with a medical consultant or professorial background; 3 charity representatives; and, 6 members of the public who had recent antenatal care experience (within 3 years) and had previously taken part in pregnancy research and self-identifying as having an interest in inclusive research strategies.

The workshop started with a context presentation, and, after discussing each indicative question in breakout rooms of 4 to 5 participants, the group met again in a plenary session for a group discussion where any question mergers, controversies, or amendments were recorded. Each breakout room included a mix of HCPs and service users, plus a facilitator. Lastly, each member of the workshop had 5 votes to cast against a final list of questions, with an option to vote for a question more than once using a dedicated online whiteboard. The facilitation team kept written documentation of the discussion on the screen so that all the workshop attendees could view how their comments were being captured. The participants also had the opportunity to add comments to the Zoom chat. During the meeting attendees were informed that all the comments would be reviewed and incorporated as a narrative summary in the report.

## Results

### National PSP survey

In the first data gathering survey, 631 individuals accepted to take part. Responses were considered valid if at least one open-ended question was answered, leaving 244 respondents. The second interim (ranking) survey had 124 respondents with 104 valid responses (ie, completed at least the first section). Valid responses were predominantly from women in surveys 1 and 2 (95% and 99%, respectively). There were more service users and partners responding to the first survey compared to HCPs (*n = *138, 57% versus *n = *103, 42%), however participation was equal in the second ranking survey. The surveys had responses from across the UK, however, London, and the Southeast region dominated the responses in both surveys (43% and 52%, respectively). A large proportion of responses came from the Southwest of England in the first survey (26%) and there were fewer responses from the Southwest, Midlands, and East of England in the second survey (10%-13%). In both surveys, approximately two-thirds of HCPs had personally experienced the antenatal care pathway in the UK. Further demographic details were not sought in the PSP survey; however, participants that met the inclusion criteria for the extended ethically approved study (outlined in Section 2) were asked for additional background information which will be reported elsewhere. A summary of the demographic information provided can be found in Table 1 below.

**Table 1.** Demographics of survey 1 (data gathering) and survey 2 (rank and choose).

	Survey 1		Survey 2	
Survey participation	Initial responses	Valid responses (%)	Initial responses	Valid responses (%)
Number, *n*	631	244 (37%)	125	104 (83%)
**Sex**	***n***	**%**	***n***	**%**
Female	233	95%	103	99%
Male	11	5%	0	0%
Prefer not to say	0	0%	1	1%
Grand total	244	100%	104	100%
**Background**	***n***	**%**	***n***	**%**
HCP	103	42%	52	50%
SU	138	57%	52	50%
Partner	2	1%	0	0%
Not answered	1	0%	0	0%
Grand Total	244	100%	104	100%
**Personal experience of pregnancy**	**HCP**	**SU**	**HCP**	**SU**
Yes, *n* (%)	66 (64)	138 (97)	34 (65)	52 (100)
No, *n* (%)	37 (36)	3 (3)	18 (35)	0 (0)
Grand total, *n*	103	141	52	52
**Location**	***n***	**%**	***n***	**%**
England, East	19	8%	10	10%
England, London, South and the South East	105	43%	54	52%
England, Midlands	20	8%	13	13%
England, North East	10	4%	9	9%
England, North West	14	6%	2	2%
England, South West	63	26%	12	12%
Northern Ireland	5	2%	1	1%
Scotland	4	2%	3	3%
Wales	4	2%	0	0%

Abbreviations: HCP = healthcare professional, SU = service user.

**Figure 2.**
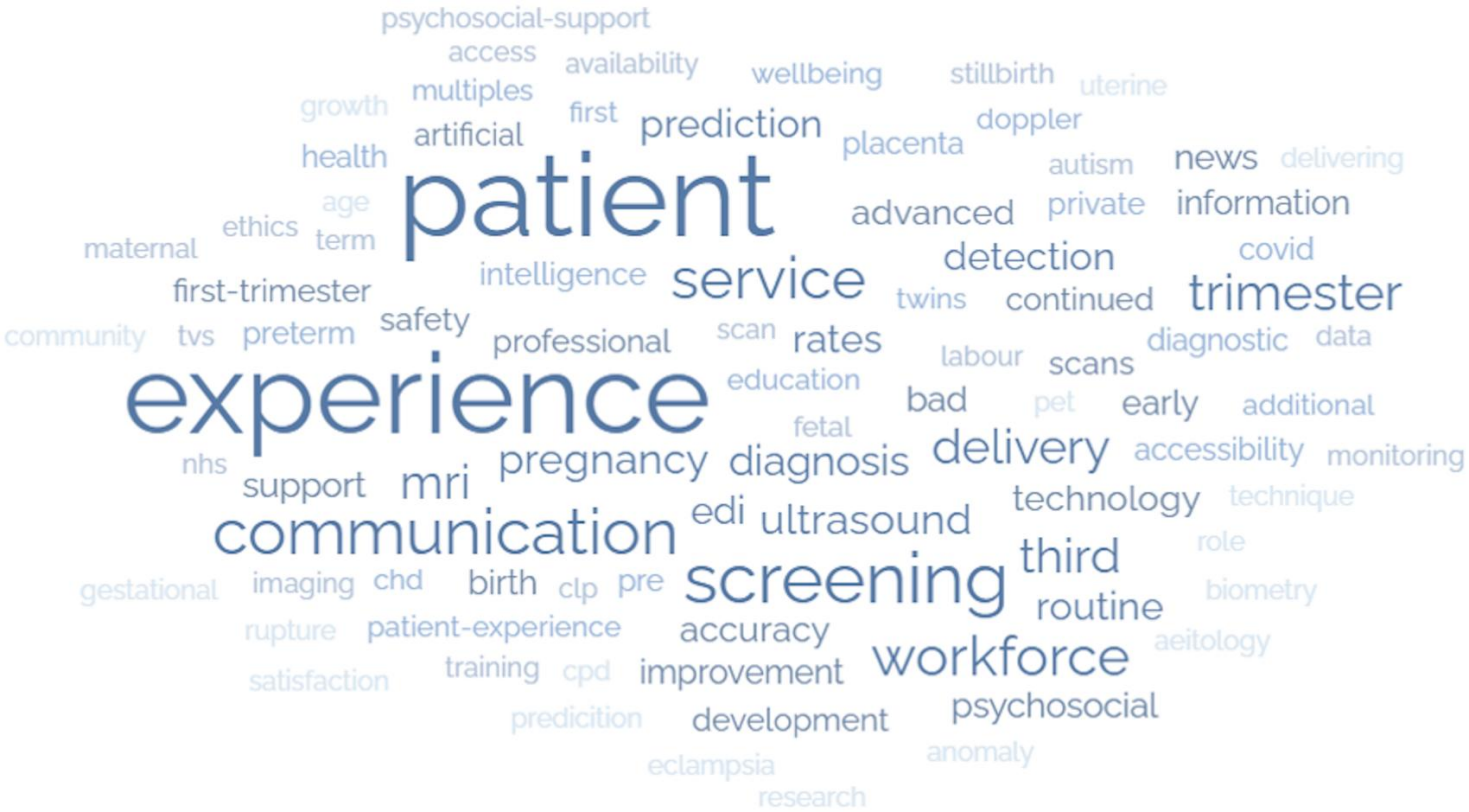
Wordcloud of qualitative thematic analysis.

**Figure 3.**
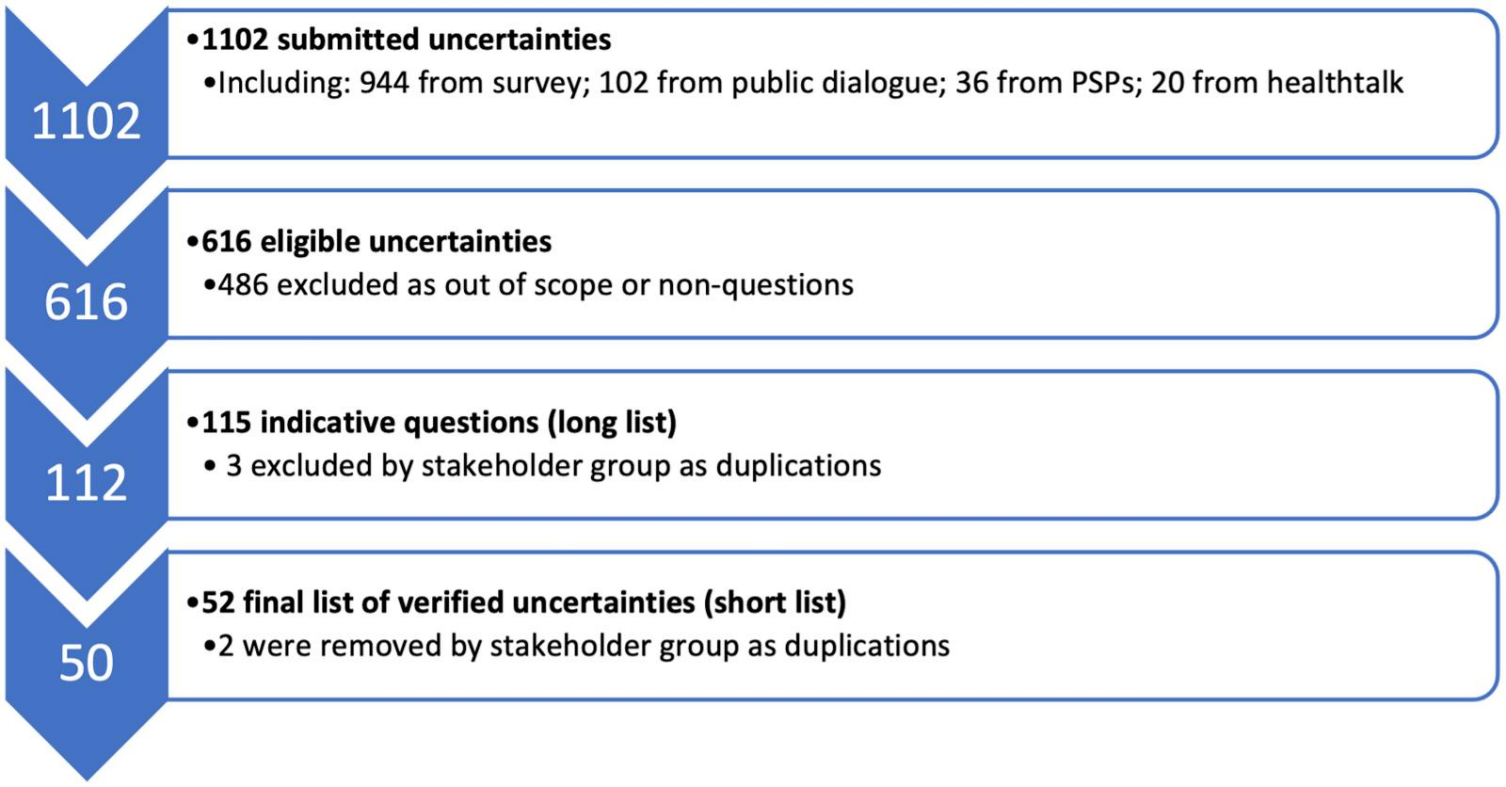
Data validation and processing flowchart to reach short list of uncertainties (*n = *50).

There were a total of 1102 submitted uncertainties, of which 944 came from the first data gathering survey and the rest from other resources that is, systematic reviews, publicly available interviews, recent public dialogue exercise.^17^ After exclusions of non-questions and obvious duplications, there were 616 eligible questions, the range of initial primary codes generated by the valid questions are presented in Figure 2. The valid questions were summarized into an initial long list of 112 indicative questions, and then consolidated into a short list of 50 indicative questions (see File S1). Both lists were discussed with the PSP stakeholder group to confirm removal of duplicates, merging of questions or alterations in wording (see Figure 3 for a flowchart of the data validation and processing).

In addition, the group analysed and consolidated the initial codes which then formed six clear top level themes, that were apparent when the long list of indicative questions were simplified to 50 questions and included:

1. Maternal and parental experience
2. Emerging technology
3. Screening, prediction, and diagnosis
4. Role of pregnancy MRI
5. Continued professional development, training and education
6. Service delivery and workforce

The second survey (interim ranking) of the 50 indicative questions, resulted in the 25 highest priority questions being selected for discussion, based on weighted ranking (number of votes by priority score. The “choose” part of the survey, where respondents were asked to choose one question per theme of their previously selected highest priorities (Table 2), resulted in one additional question, related to the role of MRI, being included in the final stakeholder workshop that were not ranked in the initial top 25 (see File S2).

**Table 2.** Highest choice question for each theme (survey 2).

Theme	Highest priority question in theme	%	Count
1 Maternal and parent experience	How can women and support partners be better supported before, during, or after miscarriage or stillbirth to reduce anxiety or stress associated scan results?	37%	27
2 Emerging technology	Can artificial intelligence, big data, or other advanced imaging technology be used to improve the accuracy of pregnancy ultrasound scans? (example priority areas may include: miscarriage, 12-week scan [nuchal translucency], 20-week scan, growth scans, heart conditions, brain conditions, cleft palate, twin pregnancies, stillbirth, rare conditions, etc.)	61%	40
3 Screening prediction and diagnosis	How can better methods/techniques/education improve prenatal detection of specific structural or developmental conditions in babies? (eg, heart conditions, cleft palate, craniosynostosis, etc.)	18%	13
4 Role of MRI^a^	What is the role of pregnancy MRI in women with raised BMI or in cases where ultrasound may be less informative?	19%	11
5 Education and CPD	How can communications training improve delivery of unexpected news during a pregnancy scan by sonographers?	44%	28
6 Service delivery and workforce	How could communication between different departments in a pregnancy care pathway be improved? that is to allow better information and support, quicker diagnosis/treatment or coordinated scans and appointments etc.	33%	22

a Additional question included in final workshop discussion.

### Final stakeholder workshop

The stakeholder workshop included 17 members: participants with a sonography professional background (*n = *4); medical consultant or professorial background (*n = *3); charity representatives (*n = *3); and members of the public (*n = *6) who had recent antenatal care experience. File S3, gives examples of the sources for the top 10 ranked questions, and Table 3 are the final top 10 questions after the workshop and includes the voting results and key discussion points.

**Table 3.** Voting results and key discussion points for top 10 priorities.

Rank	Question	Theme	Discussion points	Edit made prior to prioritization	Total votes
1	Can fetal size and growth estimation be improved?	Screening, prediction, and diagnosis	Questions around if this included training and/or technical issues	None	10
2	How can women and support partners be better supported before, during, or after miscarriage or stillbirth to reduce anxiety or stress associated scan results?	Maternal and parental experience	Generated the most discussion of all questions, centred on how inadequate communication can contribute to ongoing trauma related to loss	Helping sonographers deal with/manage parents in distress—communicating during times of anxiety or stress.	8
3	How can sonographers provide better support for people during a scan who are experiencing or who have experienced a previous high risk pregnancy or pregnancy loss?	Maternal and parental experience	Explaining history over and over can be difficult. Discussion about how this is currently communicated and what other methods might help for example, digital notification	Being aware of/acknowledging and sensitive to history/past experience.	7
4	How could communication between different departments in a pregnancy care pathway be improved? that is to allow better information and support, quicker diagnosis/treatment or coordinated scans and appointments etc.	Service delivery and workforce	Communication between departments/specialisms can help improve service experience and communication with parents	This question was merged with a similar question focused on communication of service aspects to parents.	7
5	Can artificial intelligence, big data or other advanced imaging technology be used to improve the accuracy of pregnancy ultrasound scans? (example priority areas may include: miscarriage, 12-week scan [nuchal translucency], 20-week scan, growth scans, heart conditions, brain conditions, cleft palate, twin pregnancies, stillbirth, rare conditions etc.)	Emerging technology	Examples (in brackets) were clarified and discussed before the voting.	None	7
6	What is the role of pregnancy MRI in women with raised BMI or in cases where ultrasound may be less informative?	Emerging technology	Wording accepted and clear	None	6
7	What is the public experience of antenatal scanning services and how can communication, psychological support, and information about the service be improved for parents?	Maternal and parental experience	Wording accepted and clear	None	6
8	How can communications training improve delivery of unexpected news during a pregnancy scan by sonographers?	Continued professional development, training, and education	Clarification of types of communication and how training needed to be embedded within wider training and current care pathway	None	5
9	How can recruitment and retention shortfalls in the sonography profession be addressed for high quality service provision? (including diversity of the workforce, mental wellbeing support, etc.)	Service delivery and workforce	Wording accepted and clear	None	4
10	Could further sonographer training or guidance improve patient care and satisfaction by ensuring practice is aligned with current care pathways and new technologies?	Continued professional development, training and education	Discussion about differences in implementing recommended guidelines that relate to care or use of new techniques/technologies	None	4

## Discussion

This public engagement project sought to collaboratively outline the top research priorities in antenatal imaging, through a series of surveys, data gathering exercises, stakeholder discussions and a final workshop. With approximately 650 000 births a year in the UK, requiring a minimum of 2 routine scans each, it is therefore not surprising that the themes in research gaps emphasize that sonographers play a crucial role in antenatal care and, not only the medical outcomes of the service users, but also their subjective experience.

Whereas sonographers are pivotal in the *screening* stage of the antenatal journey, it is clearer that the top research gaps must elucidate the quality of the alliance between imagers and other HCPs implicated in predictions and diagnostics. Formulating a prescriptive list of research questions is beyond the scope of this PSP but, to address the first 3 themes, we propose future study designs should assess service outcomes and usage pathways in aspects such as quality of care, inclusivity of the workforce and addressing disparities among expecting parents. A more specific starting point could be evaluating digital harmonization across services, for example, between a sonographer and an obstetrician or a midwife who may be based in wider community services or even in the same hospital. This may be a first step in identifying communication barriers between HCPs potentially affecting patient outcomes.

Strengths of this report lie in the implementation of long-standing guidelines from the JLA, the application of several stakeholders who provided the insight into the formulation of the questions as well as the selection of research priorities. Having both virtual and in-person interactions proved to be efficient and flexible in joining a wide variety of HCPs and services users. This report further exemplifies the value of coproduction in health and social care research at each step of the design. The views of HCPs and service users were arguably well represented by their equal split in numbers of respondent to our survey. Although respondents were mostly located in the South of England, the survey did reach throughout the UK. It is important to note that the national remit of this work also does not take in to account global priorities related to antenatal imaging and these priorities may differ across geographies, health service models and varying political and economic status.

Our report is further limited by responses being obtained when antenatal services were slowly reversing major changes applied in the previous 1.5 years due to the pandemic. Indeed, the generalizability of our findings would only be justified if public antenatal care services were homogeneous nationwide. This is unlikely to be the case, as trends related to discrepant outcomes are found across the UK.^38^^,^^39^ Hence, we cannot exclude that the top 10 questions may have been directly influenced by the recovery from the pandemic or variation in service, for example, in relation to communication between departments in the care pathway, psychological support for service users and training for imagers. This may also have led to research topics within the sonography/MRI care pathway being omitted and a lack of representations of all patients and service users. For example, provision towards high-risk pregnancies due to obstetric history such as miscarriage are highlighted in the top 10 questions. However, provision towards parental mental health or neurodivergence was not found, whereas these groups are suspected to require an adapted form of psychosocial support and specialized training required for imaging staff.^40^ It is possible the gaps identified here are not reflective of the research priorities for all intersections of the service users. The coproduction stage could, therefore, have included a wider diversity of users to obtain evidence of this. Additionally, some workshop participants commented that the shortlisted questions may have been too broad or overlapping in interpretation for them to be inclined to prioritize them. Hence, we find that the methodology of this PSP could be improved in future similar projects.

Future direction likely to ensue from this report, are its dissemination and promotion among clinical practitioners, research groups as well as other stakeholders. Being the first of its kind, this report is useful for researchers and may support applications for funding directed at answering the gaps identified. The authors hope that such findings communicate to patient facing support networks and third-party regulators (eg, Care Quality Commission) that the field of antenatal radiology is keen to identify the gaps in research which would improve this domain of healthcare.

## Conclusion

Whilst research activity should not be limited to the broad questions posed, the “pregnancy scanning PSP top 10” serves as a useful resource for funders and academics, and importantly, to practicing HCPs, that is, sonographers, radiographers, midwives, radiologists, and obstetricians, who aim to provide evidence for best practice within obstetric imaging services that are in line with public interest. Finally, we recommend and encourage inclusivity in research within the imaging antenatal pathway, the recognition of the multidimensionality of “high-risk” services users (previous miscarriage, high BMI, neurodivergence, mental health distress), who may require specific technical and/or psychosocial provision within and between services.

## Supplementary Material

tqaf192_Supplementary_Data
