# Supplementary material for: Setting the research priorities for pregnancy scanning: a nationally coproduced vision with expectant women, the public and healthcare professionals
Source: Br J Radiol. 2025 Aug 4;99(1180):725–32. doi: 10.1093/bjr/tqaf192 (PMC13070638; doi:10.1093/bjr/tqaf192)
Supplement: tqaf192_Supplementary_Data [file tqaf192_supplementary_data.pdf]

Supplementary file 1: Top 50 question identified

Unranked Question Number	Indicative Question and ID (ID = theme number and question number within theme)
1	TH1Q1: How can sonographers provide better support for people during a scan who are experiencing or who have experienced a previous high risk pregnancy or pregnancy loss?
2	TH1Q2: How can the scan service experience be optimised before, during or after an internal (transvaginal) ultrasound in pregnancy?
3	TH1Q3: What are the parents' perspectives (and understanding) on the ethics of collecting health data routinely for use in pregnancy scanning research?
4	TH1Q4: How can women (and support partners) who need regular ultrasound monitoring during pregnancy be better supported? (e.g. when repeat growth scans are needed for pregnancy diabetes, or other concerns about baby's health or growth)
5	TH1Q5: How can women and support partners be better supported before, during or after miscarriage or stillbirth to reduce anxiety or stress associated scan results?
6	TH1Q6: What is the role of prenatal lifestyle advice/advocacy or social prescribing referrals (e.g. stopping smoking, pregnancy nutrition or stress management) in a scan setting?
7	TH1Q7: What is the impact and benefit for parents receiving souvenir photos during antenatal scans?
8	TH1Q8: What is the public experience of antenatal scanning services and how can communication, psychological support and information about the service be improved for parents?
9	TH2Q1: Can artificial intelligence, big data or other advanced imaging technology be used to improve the accuracy of pregnancy ultrasound scans? (example priority areas may include: miscarriage, 12-week scan (nuchal translucency), 20-week scan, growth scans, heart conditions, brain conditions, cleft palate, twin pregnancies, stillbirth, rare conditions etc)
10	TH2Q2: What ethical standards need to be in place to ensure unbiased, transparent and safe implementation and use of AI in pregnancy scanning?
11	TH2Q3: How will advances in technology (e.g. AI or digital records) impact the patient/parent experience of pregnancy care? (e.g. perception of medicalisation, impact on anxiety, access to digital results)
12	TH3Q1: How can prediction models be improved to predict potential pregnancy-related conditions and then better integrated into the care pathway? (for example in ectopic pregnancies, pre-eclampsia, miscarriage, preterm birth, c-sections, stillbirth, placenta problems)
13	TH3Q2: Is there a role for routine 'detailed anatomy' scans in pregnancy before 20 weeks?
14	TH3Q3: What is the best prediction model for Down's syndrome in the era of accurate non-invasive prenatal (blood) tests? i.e. how useful is ultrasound for modern prediction models?

15 TH3Q4: How can better methods/techniques/education improve prenatal detection of specific structural or developmental conditions in babies? (e.g. heart conditions, cleft palate, craniosynostosis, etc)

16 TH3Q5: What are the advantages, limitations and challenges of a routine third trimester scan? (in the setting of monitoring reduced fetal movements, preventing stillbirth, in growth conditions, or emotional reassurance etc)

17 TH3Q6: What are the advantages, limitations and challenges of offering 3D ultrasound scans in pregnancy as part of NHS care?

18 TH3Q7: How can the wider use of existing tests, biomarkers and scans improve the prediction of pregnancy-related conditions and outcomes? (e.g. stillbirth, pre-eclampsia, neonatal death, miscarriage, etc)

19 TH3Q8: What is the structure, function and role of the placenta in pre-eclampsia?

20 TH3Q9: What are the expected growth patterns of 'small for gestational (pregnancy)-age' multiples in pregnancy e.g. twins?

21 TH3Q10: Are there any risks or safety implications to the unborn fetus undergoing ultrasound, MRI, 3D imaging?

22 TH3Q11: Can the amniotic fluid (waters) be more accurately assessed?

23 TH3Q12: Can fetal size and growth estimation be improved?

24 TH3Q13: Can conditions that manifest in later life be detected during pregnancy eg autism, adult heart disease, metabolic diseases

25 TH3Q14: What rare conditions should be screened for with ultrasound to improve detection? e.g. syndromes/genetic conditions (involving genes), or pregnancy-specific conditions like vasa previa

26 TH4Q1: What is the role of MRI scans in the diagnosis of pregnancy conditions?

27 TH4Q2: In a ‘high risk’ pregnancy setting, what is the impact of showing parents their baby's MRI images?

28 TH4Q3: Can MRI impact care management of pregnant women who have uterine fibroids or other gynaecological conditions?

29 TH4Q4: How should pregnancy MRI be used to best compliment ultrasound?

30 TH4Q5: Can pregnancy MRI provide a deeper clinical understanding of the placenta and associated conditions in pregnancy? e.g. pre-eclampsia

31 TH4Q6: What is the role of pregnancy MRI in prediction and management of preterm birth?

32 TH4Q7: How can pregnancy MRI technology and the images acquired be improved so that it can become more accurate in a wider range of pregnancy conditions?

33 TH4Q8: What is the role of pregnancy MRI in women with raised BMI or in cases where ultrasound may be less informative?

34 TH4Q9: What is the parental experience of pregnancy MRI?

35 TH4Q10: Should pregnancy MRI be more accessible and available as an imaging modality?

36 TH5Q1: In the sonography profession, how can health care education be used effectively to improve communication and patient experience when referred for hyperemesis gravidarum?

37 TH5Q2: Could a specialist sonographer role improve parent experience and/or outcomes in multiple (twin/triplet +) pregnancies?

38 TH5Q3: Could further sonographer training or guidance improve patient care and satisfaction by ensuring practice is aligned with current care pathways and new technologies?

39 TH5Q4: How effective is the consent process for ultrasound examinations?

40 TH5Q5: How can communications training improve delivery of unexpected news during a pregnancy scan by sonographers?

41 TH6Q1: What are health care professionals' views on parental perceptions of health conditions that may be diagnosed in pregnancy? (e.g. Downs syndrome, cleft lip)

42 TH6Q2: How will the future use of artificial intelligence tools impact the ultrasound workforce? E.g. reduced or increased repetitive strain injury, job satisfaction, fewer jobs

43 TH6Q3: Is there a role for pregnancy ultrasound in a community setting (e.g. health centres, GP clinics or in homes where necessary)?

44 TH6Q4: How can recruitment and retention shortfalls in the sonography profession be addressed for high quality service provision? (including diversity of the workforce, mental wellbeing support etc)

45 TH6Q5: How should parents and service users be involved in the service delivery improvements and development of standards and guidance in obstetric ultrasound to ensure practice and decision-making reflects the patients needs?

46 TH6Q6: How could communication between different departments in a pregnancy care pathway be improved? i.e. to allow better information and support, quicker diagnosis/treatment or coordinated scans and appointments etc

47 TH6Q7: What systems, policies and governance is required to support the sonographer’s role and sonographer role extension (including delivering unexpected news, consent, making referrals, treatment, interventions, service delivery)

48 TH6Q8: How does current audit practice in obstetric ultrasound improve the local and national data for the monitoring of ultrasound quality, diagnosis, detection and service delivery?

49 TH6Q9: What is the role of private obstetric scans compared to a NHS pregnancy care pathway?

50 TH6Q10: What are the barriers to effective communication between sonographers, expectant parents and/or supporting persons?

Supplementary file 2: Top 26 ranked indicative questions for the final workshop

Rank number	Question number	Theme	Question	Percent of vote for high priority
1	5	Maternal and Parental experience	TH1Q5: How can women and support partners be better supported before, during or after miscarriage or stillbirth to reduce anxiety or stress associated scan results?	82.7
2	1	Maternal and Parental experience	TH1Q1: How can sonographers provide better support for people during a scan who are experiencing or who have experienced a previous high risk pregnancy or pregnancy loss?	81.7
3	40	Continued professional development, education and training	TH5Q5: How can communications training improve delivery of unexpected news during a pregnancy scan by sonographers?	71.1
4	18	Screening, prediction, diagnosis	TH3Q7: How can the wider use of existing tests, biomarkers and scans improve the prediction of pregnancy-related conditions and outcomes? (e.g. stillbirth, pre-eclampsia, neonatal death, miscarriage, etc)	68.9
5	15	Screening, prediction, diagnosis	TH3Q4: How can better methods/techniques/education improve prenatal detection of specific structural or developmental conditions in babies? (e.g. heart conditions, cleft palate, craniosynostosis, etc)	63.3
6	9	Emerging Technology	TH2Q1: Can artificial intelligence, big data or other advanced imaging technology be used to improve the accuracy of pregnancy ultrasound scans? (example priority areas may include: miscarriage, 12-week scan (nuchal translucency), 20-week scan, growth scans, heart conditions, brain conditions, cleft palate, twin pregnancies, stillbirth, rare conditions etc)	60.6
7	46	Service delivery and workforce	TH3Q6: What are the advantages, limitations and challenges of offering 3D ultrasound scans in pregnancy as part of NHS care?	59.5

8	12	Screening, prediction, diagnosis	TH3Q1: How can prediction models be improved to predict potential pregnancy-related conditions and then better integrated into the care pathway? (for example in ectopic pregnancies, pre-eclampsia, miscarriage, preterm birth, c-sections, stillbirth, placenta problems)	57.8
9	23	Screening, prediction, diagnosis	TH3Q12: Can fetal size and growth estimation be improved?	55.6
10	25	Screening, prediction, diagnosis	TH3Q14: What rare conditions should be screened for with ultrasound to improve detection? e.g. syndromes/genetic conditions (involving genes), or pregnancy-specific conditions like vasa previa	55.6
11	16	Screening, prediction, diagnosis	TH3Q5: What are the advantages, limitations and challenges of a routine third trimester scan? (in the setting of monitoring reduced fetal movements, preventing stillbirth, in growth conditions, or emotional reassurance etc)	54.4
12	8	Maternal and Parental experience	TH1Q8: What is the public experience of antenatal scanning services and how can communication, psychological support and information about the service be improved for parents?	53.8
13	10	Emerging Techology	TH2Q2: What ethical standards need to be in place to ensure unbiased, transparent and safe implementation and use of AI in pregnancy scanning?	53.2
14	50	Service delivery and workforce	TH6Q10: What are the barriers to effective communication between sonographers, expectant parents and/or supporting persons?	51.9
15	19	Screening, prediction, diagnosis	TH3Q8: What is the structure, function and role of the placenta in pre-eclampsia?	51.1
16	44	Service delivery and workforce	TH6Q4: How can recruitment and retention shortfalls in the sonography profession be addressed for high quality service provision? (including diversity of the workforce, mental wellbeing support etc)	50.6
17	21	Screening, prediction, diagnosis	TH3Q10: Are there any risks or safety implications to the unborn fetus undergoing ultrasound, MRI, 3D imaging?	50.0

18	38	Continued professional development, education and training	TH5Q3: Could further sonographer training or guidance improve patient care and satisfaction by ensuring practice is aligned with current care pathways and new technologies?	48.2
19	4	Maternal and Parental experience	TH1Q4: How can women (and support partners) who need regular ultrasound monitoring during pregnancy be better supported? (e.g. when repeat growth scans are needed for pregnancy diabetes, or other concerns about baby's health or growth)	48.1
20	11	Emerging Technology	TH2Q3: How will advances in technology (e.g. AI or digital records) impact the patient/parent experience of pregnancy care? (e.g. perception of medicalisation, impact on anxiety, access to digital results)	46.8
21	37	Continued professional development, education and training	TH5Q2: Could a specialist sonographer role improve parent experience and/or outcomes in multiple (twin/triplet +) pregnancies?	44.6
22	20	Screening, prediction, diagnosis	TH3Q9: What are the expected growth patterns of 'small for gestational (pregnancy)-age' multiples in pregnancy e.g. twins?	44.4
23	30	Role of MRI	TH4Q5: Can pregnancy MRI provide a deeper clinical understanding of the placenta and associated conditions in pregnancy? e.g. pre-eclampsia	43.0
24	47	Service delivery and workforce	TH6Q7: What systems, policies and governance is required to support the sonographer's role and sonographer role extension (including delivering unexpected news, consent, making referrals, treatment, interventions, service delivery)	41.8
25	43	Service delivery and workforce	TH6Q3: Is there a role for pregnancy ultrasound in a community setting (e.g. health centres, GP clinics or in homes where necessary)?	40.5
26*	33	Role of MRI	TH4Q8: What is the role of pregnancy MRI in women with raised BMI or in cases where ultrasound may be less informative?	31.4
* included as a high priority question by theme				

Supplementary file 3: PSP Spreadsheet of Data for Top 10 Ranked Priorities after Survey 2

Rank number from survey 2	Question Number	Theme	Indicative Question	Example 1 of original uncertainty (source)	Example 2 original uncertainty (source)	Total submitted uncertainties (n)	service user* (n)	HCP (n)	PSP (n)	Plain language summary
1	5	Maternal and Parental experience	TH1Q5: How can women and support partners be better supported before, during or after miscarriage or stillbirth to reduce anxiety or stress associated scan results?	Could women be better prepared for the possibility of miscarriage? (miscarriage PSP)	Is it possible to have an advocate on hand or very close by when a woman has additional scans as part of fetal medicine investigations? (PSP025)	6	2	0	4	Miscarriages, stillbirths and other poor maternal outcomes are traumatic and emotional experiences for any expectant mother and partner. To reduce negative impact of this experience support is needed before, during and after the scan diagnosis.
2	1	Maternal and Parental experience	TH1Q1: How can sonographers provide better support for people during a scan who are experiencing or who have experienced a previous high risk pregnancy or pregnancy loss?	How can women who have experienced early miscarriages be better supported in scans for subsequent pregnancies? (PSP044)	How can sonographers adjust their behaviour to better support families who have experience baby loss or death and are now pregnant after loss? (PSP062)	27	23	4	0	Sonographers are often the first healthcare professional to deliver unexpected news and often during an ultrasound scan. Unexpected news can be highly emotional and delivering the correct level of support is essential at the time and also in subsequent pregnancies when attending for scans.

3	40	Continued professional development, education and training	TH5Q5: How can communications training improve delivery of unexpected news during a pregnancy scan by sonographers?	Now that the consensus guidelines for unexpected news have been implemented, in reality how have these been implemented and accepted in clinical practice? Have patients and partners noticed any improvement in the communication of unexpected news? (PSP176)	Training on how to break bad news to partners and family (HT06)	5	3	2	0	Delivering unexpected news such as a pregnancy loss requires a high level of communication skills. Education and training should help ensure sonographers maintain and develop the level of skill needed.
4	18	Screening, prediction, diagnosis	TH3Q7: How can the wider use of existing tests, biomarkers and scans improve the prediction of pregnancy-related conditions and outcomes? (e.g. stillbirth, pre-eclampsia, neonatal death, miscarriage, etc)	- the woman as a person (height, body weight, other characteristics) to make scans more accurate (PD94)	Can the wider use of existing tests and monitoring procedures, especially in later pregnancy, and the development and implementation of novel tests (biomarkers) in the mother or in early pregnancy, help prevent stillbirth? (stillbirth PSP)	2	1	0	1	Many diagnostic tests exist within healthcare, these may compliment ultrasound and other imaging techniques to develop a further understanding of fetal and maternal conditions.
5	15	Screening, prediction, diagnosis	TH3Q4: How can better methods/techniques/education improve prenatal detection of specific structural or developmental conditions in babies? (e.g. heart conditions, cleft palate, craniosynostosis, etc)	How can we use technology to improve antenatal scans to provide more detail and accurate information? (PSP048)	How can ultrasound be used to improve the detection of fetal cardiac anomalies? (PSP048)	15	7	6	2	The principal aim of an ultrasound is to detect abnormalities set within FASP guidance. No fetal abnormality has a 100% detection rate, cardiac abnormalities have a relatively low detection rate. Various methods are needed determine why detection rates are low

										and how they can be improved.
6	9	Emerging Technology	TH2Q1: Can artificial intelligence, big data or other advanced imaging technology be used to improve the accuracy of pregnancy ultrasound scans? (example priority areas may include: miscarriage, 12-week scan (nuchal translucency), 20-week scan, growth scans, heart conditions, brain conditions, cleft palate, twin pregnancies, stillbirth, rare conditions etc)	How can AI be used to reduce disparity of diagnosis and detection of anomalies? (PSP009)	In AI how will the data be used to represent those of a minority population, will there be adequate data to facilitate their care (PSP178)	36	7	3	0	Advanced in technology allow exploration of various technique to improve the detection of abnormalities. Artificial intelligence is one way of using technology to interpret and detect to greater accuracy than human.
7	46	Service delivery and workforce	TH3Q6: What are the advantages, limitations and challenges of offering 3D ultrasound scans in pregnancy as part of NHS care?	Is there a role for 3/4D ultrasound in the diagnosis of complex conditions by sonographers are part of routine screening? (PSP009)	Would having a routine 4D scan improve maternal mental health and aid bonding with the baby, thus reducing poor maternal mental health (PSP015)	20	10	10	0	3- dimensional (3D) ultrasound allows the view of the fetus in more rounded planes for example skin surfaces than conventional ultrasound. For this reason it is commonly used as a preview of the baby in private settings. However, such technology and 3D scans maybe used within routine NHS services.

8	12	Screening, prediction, diagnosis	TH3Q1: How can prediction models be improved to predict potential pregnancy-related conditions and then better integrated into the care pathway? (for example in ectopic pregnancies, pre-eclampsia, miscarriage, preterm birth, c-sections, stillbirth, placenta problems)	What are the causes of miscarriage in early pregnancy that can be predicted / possibly prevented with interventions following scan / screening? (PSP018)	How can imaging improve the prediction and fetal outcome of premature rupture of membranes? (PSP146)	28	12	13	3	Information form scans and also patient history or lab results can sometimes be used to gain an indication of how the pregnancy may develop. This question relates to more research at different stages of pregnancy to help with better prediction.
9	23	Screening, prediction, diagnosis	TH3Q12: Can fetal size and growth estimation be improved?	Are growth scans accurate for all ethnicities?	How can fetal interorbital diameter be used as a predictor of Gestational age? (PSP040)	5	1	4	0	Part of an ultrasound assessment involves obtaining well defined measurements in order to assess the fetal growth and size. However, this has an inaccuracy level. Assessment of fetal growth is essential in pregnancy and can predict fetal outcome.
10	25	Screening, prediction, diagnosis	TH3Q14: What rare conditions should be screened for with ultrasound to improve detection? e.g. syndromes/genetic conditions (involving genes), or pregnancy-specific conditions like vasa previa	Should universal screening for Vasa praevia be adopted as a screening standard (at anomaly scan)?	Picking up other conditions that they don't currently look for. (PD07)	2	1	1	0	FASP protocol suggests 11 conditions should screened for and potentially can be detected during a routine anomaly scan. However, there are many other rarer conditions, syndromes and complications which lie outside the FASP criteria and may benefit from early detection by ultrasound.
